# Data sharing and privacy issues in neuroimaging research: Opportunities, obstacles, challenges, and monsters under the bed

**DOI:** 10.1002/hbm.25120

**Published:** 2020-07-04

**Authors:** Tonya White, Elisabet Blok, Vince D. Calhoun

**Affiliations:** ^1^ Department of Child and Adolescent Psychiatry/Psychology Erasmus University Medical Center Rotterdam The Netherlands; ^2^ Department of Radiology Erasmus University Medical Center Rotterdam The Netherlands; ^3^ Tri‐institutional Center for Translational Research in Neuroimaging and Data Science (TReNDS) Georgia State University, Georgia Institute of Technology, Emory University Atlanta Georgia USA

**Keywords:** data ownership, data sharing, ENIGMA, general data protection regulation, HIPAA

## Abstract

Collaborative networks and data sharing initiatives are broadening the opportunities for the advancement of science. These initiatives offer greater transparency in science, with the opportunity for external research groups to reproduce, replicate, and extend research findings. Further, larger datasets offer the opportunity to identify homogeneous patterns within subgroups of individuals, where these patterns may be obscured by the heterogeneity of the neurobiological measure in smaller samples. However, data sharing and data pooling initiatives are not without their challenges, especially with new laws that may at first glance appear quite restrictive for open science initiatives. Interestingly, what is key to some of these new laws (i.e, the European Union's general data protection regulation) is that they provide greater control of data to those who “give” their data for research purposes. Thus, the most important element in data sharing is allowing the participants to make informed decisions about how they want their data to be used, and, within the law of the specific country, to follow the participants' wishes. This framework encompasses obtaining thorough informed consent and allowing the participant to determine the extent that they want their data shared, many of the ethical and legal obstacles are reduced to just monsters under the bed. In this manuscript we discuss the many options and obstacles for data sharing, from fully open, to federated learning, to fully closed. Importantly, we highlight the intersection of data sharing, privacy, and data ownership and highlight specific examples that we believe are informative to the neuroimaging community.

## Graphical abstract

Collaborative networks and data sharing initiatives are broadening the opportunities for the advancement of science. However, data sharing initiatives are not without their challenges, especially with new laws that may at first glance appear quite restrictive for open science initiatives. This article discusses the opportunities, challenges, benefits for sharing neuroimaging data, including the missed opportunities in not providing participants greater say in opportunities for data sharing initiatives.

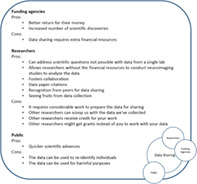

## 1 INTRODUCTION

The word “data” is the plural form of the Latin word *datum*, meaning “a thing given.” This definition is very appropriate in human subjects research, as participants are giving (actually entrusting) researchers something of themselves, which researchers in turn collect and store (as data) to be used to address important questions in science. In many cases, these “things given” by the participants result in no direct benefit to the individual themselves, but there lies the hope that it may help others. Indeed, within the larger scope of medical research, the use of these “things given” (data) has resulted in immense progress over the past century in preventions, cures, and in the treatments of a myriad of conditions. Just two widely known examples include the links between smoking and cardiovascular disease (Ambrose & Barua, 2004) and cancer (O'Keeffe et al., 2018); and the links between low folate during pregnancy and the increased risk of neural tube defects (Blom, Shaw, Den Heijer, & Finnell, 2006). However, these are just a drop in the bucket of how medical research has resulted in improving the health and well‐being of the population. Translating research for the benefit of the population would be very challenging without participants entrusting researchers with their data.

In addition to the dramatic progress over past century in the manner in which data has been used, there have also been considerable advances in the methods of study design, data collection, and data analyses and importantly, dramatic changes in the ethics of human subject data (Leonelli, 2016; Nichols et al., 2017). Recent advances have not only involved the creation and improvement of treatments and preventive care, but also the cessation of treatments or programs that either do not work, or worse, are harmful to patients. Thus, these “things given” have resulted in much that is good and very beneficial for others. At the same time that study participants are providing their data for research, there has also been dramatic progress over the last decade with researchers beginning to “share” these “things given” (data) with other researchers. Within human subjects research, large data sharing or data pooling initiatives have been especially prominent in the fields of genetics, neuroimaging, and the combination of the two (Poldrack & Gorgolewski, 2014; Poline et al., 2012; Thompson et al., 2014). These data sharing initiatives help advance the process of scientific discovery through increasing sample sizes, which allows for greater precision and the ability to measure smaller effects, although smaller effects can also be associated with either smaller biological effects or potentially confounding factors (Smith & Nichols, 2018). Larger sample sizes also offer the ability to parse the considerable heterogeneity of the population into more homogeneous groups. This may be beneficial in increasing the specificity of the underlying neurobiology of specific characteristics or illnesses or to develop more individualized reference models (Manrai, Patel, & Ioannidis, 2018). Larger sample sizes will offer the opportunity to apply more sophisticated statistical models to the data (Bzdok & Yeo, 2017), since most biological processes have non‐linear and stochastic mechanisms (White, 2019). In addition, data sharing initiatives open the door for reproducibility, replication, and increased transparency (Milham et al., 2018).

Typically when you give something away, that which was given is no longer yours, but rather belongs now to the person who received the gift. Researchers and universities often hold the view that the data “belongs” to them, they have received the gift and are now the true owners of the data. Funding agencies in the United States consider institutions to be the owners of the data. However, the question regarding ownership of data is quite complex and, as discussed below, recent laws such as the European Union (EU)'s general data protection regulation (GDPR) are giving more rights to the individuals who are participating in studies (GDPR, 2016).

The GDPR went into effect on May 25, 2018 and involves data privacy laws involving the storage, transfer, and sharing of data, both within and outside the EU and extending to the European Economic Area. The GDPR places greater responsibility on institutions to safeguard the privacy of personal data, such as assuring that there is a data controller to monitor data security. In addition, the GDPR's “Privacy by Design” requires that the safeguarding of data should be discussed and implemented during the design phase of the study. Contrasting the GDPR with laws in the United States, the GDPR provides regulations that extend broadly to all personal information, whereas personal information in the United States falls either under the Common Rule or the Health Insurance Portability and Accountability Act (HIPAA). These are described in more detail later.

There has been a paradigm shift over the last decade with respect to data ownership, partially driven by recent events in which personal data was used without consent of the individuals for monetary or political motives. These events that received considerable media coverage highlight the importance of big data (Rosenberg, Confessore, & Cadwalladr, 2018). However, these recent events also highlight the importance of the conscientious and ethical use of human subjects data, fostering a culture of data sharing for the benefit of the population, while also protecting the privacy of the individuals who are participating in the studies. Different institutions and countries have differences in their approach to balancing data protection and data sharing and researchers need to work within the borders of the laws of the countries where they reside. Within this context, it is the goal of this manuscript to provide information on the opportunities, obstacles, and challenges related to sharing human subjects data. While we focus specifically on the sharing of neuroimaging data, many of the points discussed can extend to other types of data elements. One key element present in privacy laws, including the GDPR and the HIPAA, involves the right of the individual to make decisions regarding their own data. Within the context of obtaining thorough and transparent informed consent/assent from our participants, we discuss whether certain regulations, such as the GDPR and HIPAA are truly monsters, or merely monsters under the bed.

## 2 FULLY ANONYMIZED VERSUS DE‐IDENTIFIED DATA

Within the framework of privacy protection, the degree of anonymization of the data is an important consideration and thus is an aspect incorporated in privacy regulations. Different rules apply to data, which are dependent on whether the data is considered personal data, fully anonymized or de‐identified. Fully anonymized data has all personalized data removed, is given a separate identification code, and the key between the fully anonymized dataset and any path back to the original data is deleted such that it would be extremely difficult to trace the data back to an individual. However, depending on the type and amount of data, machine learning algorithms could, within a specific probability distribution, trace back to a specific individual.

Typically, fully anonymized data can be shared without the consent of an individual. However, there are a set number of criteria that need to be met before data can be considered fully anonymized. These include taking actions to prevent the possibility of tracing, linking or deducing individuals from the data. Each variable or combination of variables that could reasonably be used to identify an individual should be taken into account. For example, low rates of specific ethnic minorities in combination with other variables, such as age and gender, could be used to re‐identify individuals. Also, more rare medical conditions coupled with other demographic features could be used to re‐identify individuals. Within this context, there has been recent concern that large datasets with multiple variables cannot truly be anonymized. By merging with other large databases, algorithms can predict within a certain error margin, whether a specific dataset belongs to a certain individual. Thus, for some large datasets it may be best consider the dataset in the de‐identified category when large numbers of variables will be shared.

Within the GDPR a clear distinction is made between personal data, de‐identified data, and fully anonymized data. Personal data refers to data that can directly define the identity of an individual, such as the name, date of birth, or the address of the individual. Within the HIPAA protected health information (PHI) is defined as “individually identifiable health information.” This includes demographic and data related to: (a), the individual's past, present or future physical or mental health or condition; (b), the provision of health care to an individual; or (c), the past, present, or future payment for the provision of health care to an individual and that identifies the individual or for which there is a reasonable basis to believe can be used to identify the individual (45 C.F.R. § 160.103). Examples of PHI are individual's names, birth dates, and genetic information.

De‐identified data means that the personal data is stripped from the dataset and the individuals are given a unique identification number, that is, the age of an individual is provided without the date of birth. However, for de‐identified data a key remains which can be used to link the de‐identified data back to the personalized data.

The data can be considered de‐identified when identifiers are replaced by artificial identifiers, so that the data cannot be linked to individuals by third parties. The possibility of encryption remains, the host researchers/data managers are allowed to have the key, which serves as the link between data and individuals. Importantly, the receivers of the data being shared also play a key role here, because they should agree to not attempt to re‐identify individuals, and, as such, terminology along these lines should be included in the data use agreement.

## 3 PRIVACY

Privacy concerns have only increased since the initial cautionary tales of the Netflix competition in which competitors inadvertently re‐identified individuals from anonymous datasets (Netflix Prize Privacy Concerns—https://en.wikipedia.org/wiki/Netflix_Prize#Privacy_concerns) and the example of genetic *reidentification* from datasets anonymized per NIH guidelines (Cassa, Wieland, & Mandl, 2008; El Emam, 2011; Homer et al., 2008). Privacy regulations are rapidly changing, including the GDPR, the California Consumer Privacy Act (CCPA), and policy restrictions across Asian countries. Projects such as the decentralized internet (Simonite, 2018) and differential privacy, such as used by Apple (Bhowmick, Duchi, Freudiger, Kapoor, & Rogers, 2018) and the 2020 U.S. Census (Census, 2020: Data Protection and Privacy Program) have entered public discourse. Differential privacy involves adding characteristic noise, often noise fitting a Laplacian distribution, in order to prevent the re‐identification of individuals (Dwork & Roth, 2013; Dwork & Smith, 2010). However, for group analyses with large sample sizes, the noise will be filtered out as the residuals.

Examples of re‐identification, such as highly accurate identification via facial reconstruction (Schwarz et al., 2019) and machine learning identification from generative models (Rocher, Hendrickx, & de Montjoye, 2019) challenge the technical and legal adequacy of the de‐identification release‐and‐forget model, spurring calls for additional privacy guidance (Morris, 2019). Other issues specific to neuroimaging data, such as personal identifiers in the DICOM header, should be removed prior to data sharing. Generally shared de‐identified or anonymized data is downloaded from a hosted site, whether the servers are located at a specific university or in the cloud. It is possible for researchers to obtain some study data, that is, Human Connectome Project data, via an encrypted hard drive that is mailed. However, given the sheer quantity of data available, image processing for large studies will require supercomputing facilities, which may include commercial cloud‐based facilities. With the proper safeguards and data agreements, cloud‐based computing will be equally as safe as an encrypted hard drive with a strong password that is behind locked doors.

In light of the possibility to re‐identify individuals based on the facial reconstruction from high‐resolution structural MRI data, there have been a number of software packages that are able to “de‐face” MR images (Bischoff‐Grethe et al., 2007; Milchenko & Marcus, 2013). Thus, for data sharing of high‐resolution structural MRI images it is important to first remove or blur the surface‐based features in the images. While programs that remove the possibility of re‐identify individuals based on their surface anatomy, they may reduce the image quality for downstream pre‐processing algorithms (de Sitter et al., 2020).

Separate from structural neuroimaging data, other neuroimaging modalities (EEG, magnetic resonance spectroscopy, magnetoencephalography (MEG) do not lend themselves to easily identifying individuals. The exception would be in the case of artifacts, such as a specific seizure disorder in EEG data which could be then coupled with other data to potentially identify an individual. The spatial resolution of both diffusion tensor imaging and functional MRI is continuously increasing, which many allow for facial characteristics to be identified and thus these high‐resolution DTI and fMRI images should also undergo defacing. While there is support that individuals have characteristic patterns of functional brain connectivity, known as functional connectome fingerprinting, these have not been used to identify individuals (Finn et al., 2015). Finally, in our longitudinal study of child development (White et al., 2017), we give children several photos of their brains (i.e., sagittal midline slice from the structural MRI) following their session, of which we have learned that some of the children have placed on social media. Thus, even removal of facial features from an MRI scan may not completely ensure privacy.

## 4 MISSED OPPORTUNITIES

Within neuroimaging there are considerable missed opportunities for data sharing: thousands of studies with data collected from valuable populations did not include data sharing language within their consent forms and some IRB and medical ethics committees are refusing to allow these data to be shared. In general, and across most countries, consent from the participants is necessary prior to the sharing of de‐identified data. While it may be possible for researchers to design their study and inclusion criteria to include only those participants who are willing to share their data, for clinical studies, this may result in a selection bias. However, this same selection bias would be present when only data with consent to share data, is shared. Thus, especially for clinical studies, an indication of the representativeness of the participants included in the data sharing initiative should be provided. The representativeness can be illustrated by comparing the demographic and clinical information of those who chose not to share their data compared to those who are willing share their data. While this will provide an indication of representativeness between those willing and not willing to share their data, it does not account for representativeness as a result of potential biases during the inclusion phase of the study (i.e., selection bias).

NIH program officers have raised concerns regarding re‐anonymization attacks (Narayanan & Shmatikov, 2008; Ravindra & Grama, 2019), the importance of security‐hardening of software tools, and privacy protection. Entering a data use agreement (DUA) can help mitigate these issues, but setting up a DUA is often a cumbersome process, requiring multiple agreements (one per site and sometime even one per researcher, including institutional sign‐off), discouraging potential users and still providing no more than a trust‐based protection of the data. Nevertheless, in spite of the obstacles, many forms of data sharing are taking place and the benefits of these efforts have been seen (Milham et al., 2018).

## 5 DATA OWNERSHIP

Data sharing is intimately tied to data ownership. However, the question of who is the actual owner of research data is complex; yet understanding this question is crucial from the perspective of data sharing. Whoever owns the data has control over the data, its dissemination, and the timing of dissemination (Fishbein, 1991). There are many parties who stake a claim for ownership; including academic institutions, researchers, funding agencies, and journals that are more and more requesting that the data supporting the articles be uploaded (Cleary, Jackson, & Walter, 2013). In many cases, both in Europe and North America, it is the academic institutions that claim ownership of data from sponsored research projects (Alter & Gonzalez, 2018). In many cases of government sponsored projects, that is, the U.S. National Institutes of Health, are considered the owners of the data. With the funding of the sponsored project, the academic institutions are then contracted to collect, clean, and to serve as the custodians of the data (Alter & Gonzalez, 2018). The university agrees to comply with specific regulations regarding the ethical collection, storage, sharing, and use of the data. The last decade has seen a paradigm shift with a number of federal government funding institutes (i.e., the National Institutes of Drug Abuse (NIDA) and the National Institutes of Mental Health (NIMH)) have laid requirements for data sharing for research that they fund. For example, the perspective of the NIH for nearly two decades has been that “all data should be considered for data sharing” (NIH, 2003). Researchers submitting applications have yearly direct costs greater than $500,000 are required to submit a data sharing plan, with the release of the data coinciding with the publication of the main findings of the study. Similarly, the EU**'**s Human Brain Project also has a major component involving data sharing. Under the recent Horizon 2020 call, as far as possible, research data should be made available to “access, mine, exploit, reproduce, and disseminate (free of charge)” research data (European‐Commission, 2017).

There has been little discussion in the literature of the participants themselves being the true owners of their research data. Yet one of the strongest messages inherent in the EU's GDPR is that individuals have much more control of their own data. This is best highlighted in the GDPR law that entails the individuals to have the “Right to be Forgotten.” The “Right to be Forgotten” essentially means that an individual can request that their complete paper and electronic research history be “erased” for a specific organization (EU, 2018). When an individual participant living within the EU invokes their “Right to be Forgotten,” any personal and de‐identified data are then erased or destroyed. It could be argued that if an individual has the right to have their data removed or destroyed, that they are the “true owners of their data.” However, the GDPR “Right to be Forgotten” does not apply to any fully anonymized data that has been released. Fully anonymized data would not contain the coded link, which would allow the data to be traced back to a specific individual.

Understanding who are the rightful owners, and who are the custodians of the data is beneficial to know how we go about data sharing. In most cases, de‐identified human subject's data, including neuroimaging and its associated meta‐data, cannot be shared without adequate consent that specifically states that the data can be shared. However, it is the researchers who write the consent forms and thus can ultimately control, to some extent, the opportunities for data sharing. A consent form written that precludes the option for data sharing dramatically limits the ability for sharing to occur, although if it's possible to fully anonymize the data, then sharing is possible in most situations. Thus, it is important that the opportunity for data sharing be given to the participants via consent, and when applicable, assent, so that they can make the decision whether they, with the optimal data protection under the law, want to share their data or not.

Under some laws individual participants can request their own data and personally share it. However, this would require considerable organization and knowledge of how the data are organized. That said, mechanisms are emerging, such as “Open Humans” (Tzovaras et al., 2019) that allow research participants, after obtaining their own data, to allow it to be uploaded to a site in which the participants receive requests when researchers would like to access their data to address specific questions. Initiative such as “Open Humans” are highlighting the potential paradigm shift related to the ownership of personal data moving in the direction of the owners being those from whom the data were originally derived. While the topic of data ownership is complex, it is a crucial element that should be discussed in the context of data sharing.

## 6 OPPORTUNITIES FOR DATA SHARING

The brain is a highly complex organism housing billions of neurons and trillions of synapses that have the ability to orchestrate a beautiful symphony of social, cognitive, and emotion functions. Within this backdrop, there is no question that it takes teamwork to understand the brain from the sub molecular to the gross anatomical level. This is supported by the recent increase in both large scale studies that make the data openly available to researchers and consortia which pool many smaller studies for either meta‐ or mega analyses.

The fields that have been at the forefront of data sharing or data pooling initiatives are those fields in which (a) data can be easily harmonized; and (b) large sample sizes are necessary and potentially available to address specific questions. Thus, it is not surprising that within medicine, it is the field of human genetics that has been at the forefront of these initiatives, followed closely by the field of neuroimaging. The combination of neuroimaging and genetics, coined “imaging genetics” has also emerged, with the Enhancing Neuro Imaging Genetics through Meta Analyses (ENIGMA) consortium playing a leading role in this initiative (Thompson et al., 2014). Fields such as epidemiology, which also benefit from larger sample sizes, lag behind in data sharing initiatives partly due to the complexities in harmonizing the different approaches to measure environmental variables (Ehrenstein, Nielsen, Pedersen, Johnsen, & Pedersen, 2017; Fairchild et al., 2018).

The opportunities for data sharing can best be portrayed in those studies or initiatives that have been very successful, with success being defined as contributing positively to the advancement of knowledge. One well known and successful approach for data sharing within a collaborative network involves the ENIGMA consortium (Thompson et al., 2014). In the ENIGMA model, the approach which allows the largest participation involves sharing pre‐ and post‐processing analysis scripts with the group. Results from each participating site are then returned to the site leading the analysis in order to conduct a meta‐analysis (e.g., Kelly et al., 2018). This provides a powerful way to leverage data from around the world and has received wide adoption by the community. Moreover, performing analyses at a centralized site has the benefit of being able to work within local or regional restrictions, whether defined by law or by the facility. However, such meta‐analytic approaches are limited to low dimensional analyses (such as volumetric analyses) and do not yet enable the use of voxelwise, surface‐based, or iterative machine learning analyses (i.e., those that perform iterative analyses using the entire dataset). To accomplish the latter, for the subset of sites that are able to have their data centralized, data can be pooled in order to perform mega‐analyses (Boedhoe et al., 2019).

There are also data sharing initiatives in which neuroimaging data collected by many different studies is retrospectively made anonymous and pooled. Examples include the Autism Brain Imaging Data Exchange (ABIDE‐I and II)(Di Martino et al., 2014, 2017), 1,000 Functional Connectomes (Biswal et al., 2010), Consortium on Reliability and Reproducibility (Zuo et al., 2014), the REST‐meta‐MDD consortium (Yan et al., 2019) and the Healthy Brains Consortium (O'Connor et al., 2017).

Funding agencies have also been instrumental in pushing for and funding a number of large studies of which data sharing is a key element. From the United States the most common include the Human Connectome Project (Van Essen et al., 2012; Van Essen et al., 2013), Baby Connectome Project (Howell et al., 2019), Alzheimer's Disease Neuroimaging Initiative (Mueller et al., 2005), Longitudinal Study of Adolescent Brain Cognitive Development or the ABCD Study (Casey et al., 2018), MIND Clinical Imaging Consortium (Gollub et al., 2013), COBRE (Aine et al., 2017), Pediatric Imaging, Neurocognition, and Genetics Study (Jernigan et al., 2016), Philadelphia Neurodevelopmental Cohort (Satterthwaite et al., 2014), and Infant Brain Imaging Study (Hazlett et al., 2017); from the United Kingdom the UK Biobank has the goal to release neuroimaging data from 100,000 participants (Miller et al., 2016), and the EU Human Brain Project (Markram, 2012) has data sharing as a key element of the grant. The NIMH currently mandates data sharing (with an institutionally signed DUA (Miller et al., 2017)) for almost all funded studies (there are some notable exceptions to this in the case of extremely sensitive data which might lead to the ability to re‐identify an individual).

## 7 A SPECTRUM OF SHARING

Data sharing of neuroimaging data can be considered to lie on a spectrum; ranging from fully open to completely closed. While the “open science” philosophy typically suggests that researchers should share as much data as possible, sharing can also be done on a smaller scale, depending on the goals. There is a broad‐spectrum of goals for which data can be shared. At one end of the spectrum data can be shared solely for reproduction (i.e., sharing only the data and code necessary to rerun the analyses to reproduce the results). In the middle of the spectrum, a subset of data can be shared that allow others to replicate findings from other studies (i.e., re‐running analyses). Finally, on the other end of spectrum is sharing all data obtained from a study. The latter allows researchers to address questions that have not been addressed before.

Table 1 lays out the trade‐offs that are present in the existing spectrum of data sharing. At one end is the sharing of peak coordinates. These are often extracted from existing manuscript tables, but may also be provided for specific individuals to provide more accurate information. This enables meta‐analytic approaches to be performed by combining experiments and studies (Fox & Lancaster, 2002). However, regions that did not reach statistical significance in the original analyses will not be included in this meta‐analytic approach. The next level is to share unthresholded (Gorgolewski, Varoquaux, et al., 2016) or network maps (Muetzel et al., 2016), which allows for voxelwise or connectivity analyses to be done even for regions that did not achieve significance in the original study. Multivariate and other advanced analytic approaches have the ability to extract a remarkable amount of information from these highly distilled features, for example, intrinsic networks can be captured from covariation among individual datasets (Calhoun & Allen, 2013; Smith et al., 2009). However, both of these approaches, though more informative and useful than peak results, still provide relatively low information relative to the raw data (Calhoun, 2015). In addition, these approaches involve retrospective storage of completed studies and do not allow for novel subject‐level models to be run on the time series data.

**TABLE 1:** A sampling of sharing approaches and their trade‐offs

What is shared	Centralized full data	Centralized individual features	Voxel‐based and machine learning	Information content	Compute load	Custom subject‐level models	Privacy
Nothing	No	No	No	None	None	No	Highest
Privatized intermediates (e.g., COINSTAC [Plis et al., 2016])^a^	No	No	Yes	High	Med‐low^b^	Yes	Higher^c^
Intermediates (e.g., COINSTAC [Plis et al., 2016])	No	No	Yes	High	Med‐low^b^	Yes	High^c^
Group coordinates (e.g., Brainmap [Fox & Lancaster, 2002])	No	No	Yes	Low	Low	No	High^d^
Features (e.g., dataShield [Wolfson et al., 2010]_	No	Yes	Yes	Med‐high	Med‐low	Yes	Med‐high^c^
Data (temporarily) (e.g., ViPAR [Carter et al., 2016])	Yes (private)	Yes	Yes	Med‐high	Med‐high	Yes	High^c^
Group maps (e.g., neurovault [Gorgolewski et al., 2016])	No	No	Yes	Med‐low	Med‐low	No	High^d^
Meta data (e.g., ENIGMA [Thompson et al., 2014])	No	No	No	Med‐low	Med‐low	Yes	Med
Mega data (e.g., ENIGMA [Thompson et al., 2014])	Yes	Yes	Yes	Med	Med	Yes	Med
Preprocessed data	Yes	Yes	Yes	High	High	Yes	Med
NIfTI data	Yes	Yes	Yes	High	High	Yes	Low
DICOM data	Yes	Yes	Yes	High	High	Yes	Low
Everything	Yes	Yes	Yes	Highest	Highest	Yes	Lowest

^a^ One can use decentralized algorithms which also include additional privacy protection by, for example, adding structured noise to the derivatives before they are sent to the aggregator (e.g., differential privacy).

^b^ Because COINSTAC preprocessing for a given site can be pre‐computed once, the computational demands for subsequence analyses can be much lower (e.g., if one wants to incorporate a remote large *N* dataset with a local smaller *N* dataset).

^c^ Derivatives are privately aggregated.

^d^ It has been shown that in multiple cases, even group averages can reveal unanticipated information about the individual.

The next level of sharing involves building consortia to analyze previously collected data as a group, often without sharing of the raw data. ENIGMA consortia (Thompson et al., 2014) have been highly successful in creating a culture of sharing built primarily around distributing a common set of scripts which are run locally. The results run locally (e.g., analyses involving volumetric MR data) are then shared for centralized meta‐analysis. This edition of Human Brain Mapping has multiple examples of this form of data sharing. In some cases, if allowable, raw or preprocessed data can also be shared, these can then be used for mega‐analyses.

Pooling results for meta‐analyses has the major advantage that data is analyzed locally and thus it is not necessary to share individual data. Moreover, it does not require advanced analysis methods to account for clustering‐effects within cohorts. However, sharing of the individual datasets can be extremely beneficial for numerous reasons, including increasing sample size (Button et al., 2013), better performance (Boedhoe et al., 2019), greater flexibility in controlling for confounders, and the ability to parse heterogeneous groups to better understand the underlying neurobiology. While certain analyses cannot be applied to small datasets, pooling these smaller datasets expands the opportunities to address specific questions and to assess the replicability of the findings.

There are also some drawbacks of pooling datasets for mega‐analyses. Differences in data‐acquisition protocols and MR platforms introduce noise in the data. However, from a clinical perspective, it is critical that findings are robust enough to be detectable across scanners and protocols. When comparing results of meta‐ and mega‐analyses, the two are fortunately quite similar (Debray et al., 2015). In addition, iterative meta‐analyses can be identical to mega‐analysis (Sarwate, Plis, Turner, Arbabshirani, & Calhoun, 2014). However meta‐analysis are limited in that it is inefficient to add additional features that were not originally included in the distributed scripts to the different sites. Further, meta‐analyses do not allow iterative approaches that require access to the first level data. Beyond this, mega‐analyses have the advantage of an increased power to detect differences, which is especially important when there are non‐significant associations at individual sites (Boedhoe et al., 2019). If certain associations reach sub‐threshold significance at individual sites, they will not be taken into account in the pooled meta‐analysis, whereas in a mega‐analysis, those associations might be discovered, simply because of the increased power.

Approaches for decentralized sharing provide a way to “thread the needle” between privacy and openness. Approaches like data SHIELD (Wolfson et al., 2010) enable analysis of centralized pre‐computed features and another approach called ViPAR (virtual pooling and analysis of research data) leverages federated databases to provide temporal pooling of the actual data for analysis (Carter et al., 2016). The collaborative informatics and neuroimaging suite toolkit for anonymous computation (COINSTAC [Plis et al., 2016]; https://github.com/trendscenter/coinstac) tool and approach goes a step further in offering fully decentralized (and potentially privatized analysis), allowing the data to remain local at the site of collection, by leveraging local compute resources for each site's data. This allows researchers to draw conclusions from large scale data without the need to have full control over the samples or aggregating them in a central place. An ongoing project (http://grantome.com/grant/NIH/R01-MH121246-01) is focused on combining the ENIGMA and COINSTAC approaches together, offering a powerful approach that leverages a large and active consortium with a decentralized analysis approach that offers advanced and high‐dimensional approaches to data that is unable to be centrally shared.

Decentralized analysis such as COINSTAC provide a way to offer access to datasets that are not currently shareable due to regulatory or other concerns. However, another important use case is the ability to link external data sources (e.g., a large curated repository of data) to local data without requiring a huge amount of local storage. The current “big data in neuroscience” era has led to, in some cases, an “analytic bottleneck,” with some groups being unable to leverage the necessary compute resources, despite the availability of cloud based analytic workbenches and repositories such as NDA, brainlife.io, Open NEURO, COINS, and many others (Eickhoff, Nichols, Van Horn, & Turner, 2016). Often there is a need to compare across datasets that are not centralized, but do allow for common references to be rapidly updated and used and to enable these data to be quickly combined with (potentially unsharable) local data. Assuring that the shared data has the optimum data quality, or including metrics that allow users to understand the underlying quality of pre‐processed images (Esteban et al., 2017; White et al., 2018) is important to reduce noise‐related variability and to increase power (Zuo, Xu, & Milham, 2019).

At the other end of the spectrum are fully open approaches mentioned earlier that share the preprocessed data, NIfTI files (avoiding potential privacy issues included in the DICOM file headers) or the DICOM files. This is the best option for research groups that focus on creating novel neuroimaging methodologies and require the raw DICOM or NIfTI neuroimaging data, as they will need software and computational power (i.e., GPUs) to run their algorithms). An early example of an fully open approach is the OpenfMRI Project (Poldrack et al., 2013), which provided an open dissemination of task‐based functional neuroimaging data. OpenfMRI has since been depreciated and has migrated to OpenNEURO (openneuro.org), which provides a platform for sharing not only MRI data, but also other imaging modalities. However, sharing data within OpenNEURO has the requirement that, following a 36‐month grace period following the first successful analysis of the data, the data will be become publicly available under a Creative Commons (CCO) license. Thus, under some regulations and certain countries, the data would need to be fully anonymized prior to being uploaded.

While there are many challenges of data sharing, sharing data alone is often not sufficient. Neuroimaging data can be highly complex and different groups have traditionally come up with their own approach to naming and storing data. However, the combination of the complexity of neuroimaging data, coupled with data sharing can result in groups spending a considerable amount of time becoming acquainted with how the data is structured. Thus, the creation of standardized approaches for naming and storing data, such as the Brain Imaging Data Structure (BIDS) (Gorgolewski, Auer, et al., 2016), is becoming increasingly adopted in the neuroimaging community. BIDS provides a mechanism to organize both NIfTI image and metadata in a uniform structure (both a uniform tree structure, naming of the data elements, and the coding of metadata) across datasets. The utilization of standardized approaches can dramatically reduce the time necessary to understand the nature of the data and to reduce the number of errors due to misunderstandings surrounding the data. In addition, increasingly more databases, such as OpenNEURO (Botvinik‐Nezer, Iwanir, Poldrack, & Schonberg, 2019), and tools for validation and data analysis packages are nested within the BIDS‐format, creating a greater incentive to be used by future researchers.

When the goal of sharing is reproduction, it is important to share not only the data, but also the scripts used to analyze the data. Pure reproduction can only be established with detailed information on the coding of variables, the approach to missing data, and how the analyses were performed. In these cases, it may be important to share not only the data that has been used for the analyses, but also the data that was excluded from the analyses. While not optimal, new techniques provide algorithms that can be used to simulate data similar to the data used in the specific studies (Shepherd, Peratikos, Rebeiro, Duda, & McGowan, 2017). This simulated data can then be used for other researchers to run the scripts on the simulated data, without gaining access to the actual data.

Sharing of scripts/code is good, but it is also not sufficient. Code is often complex and is constantly undergoing changes and updates. Versioning approaches like GitHub can help with tracking the versions used, but beyond this it would be beneficial to have tools that would enable recording the full provenance of the analyses, including code. Both code and data could be stamped with a unique doi, for example, including information about the computer used and each process having a timestamp. Even just the analysis pipeline is incredibly complex, initiatives like the neuroimage data model are working to try to incorporate standardized provenance tracking into the major analysis packages (Dinov et al., 2010; Keator et al., 2013).

## 8 OBSTACLES FOR DATA SHARING

**FIGURE 1:**
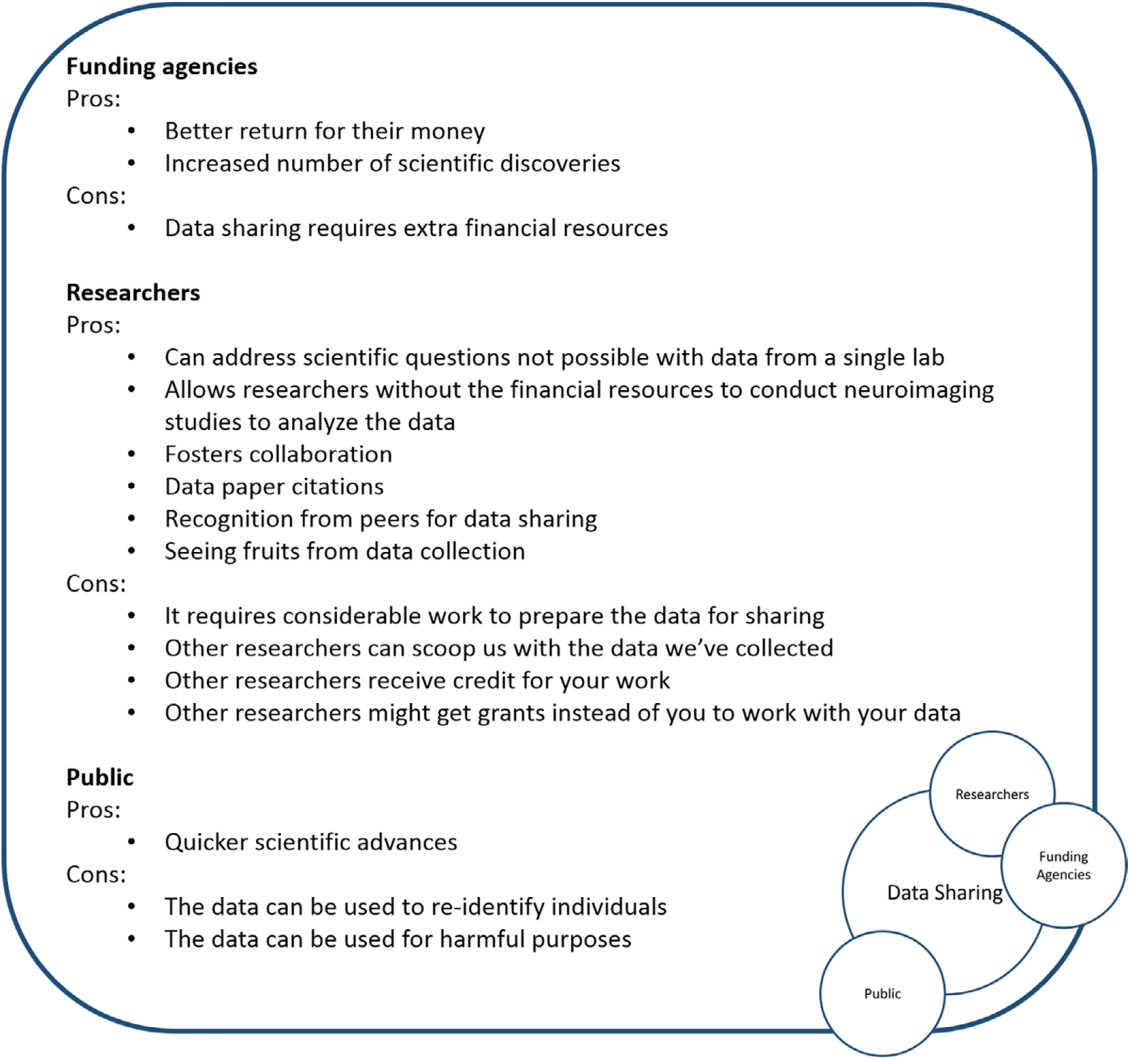
The pros and cons of data sharing from the perspective of funding agencies, the public, and researchers

Funding agencies are nearly unanimous in their support of data sharing. With appropriate consent and, if necessary, assent, participants can determine whether they wish to have their data shared or not. Thus, the greatest obstacle for data sharing lies not with the participants, nor with the funding institutions, nor with legal aspects related to data sharing, but rather with the researchers. One obstacle for researchers is that it requires considerable work to do it well, and there is currently very little credit or compensation for data sharing. Shared data needs to be carefully curated and described in ways that other researchers can use the data properly (Leonelli, 2014), which is above and beyond the standard work load. Research careers are primarily evaluated on the number, quality, and impact of papers published; and the acquisition of grant funding, where the acquisition of grant funding being dependent on the number, quality and impact of the publications. There are a number of valid concerns raised by researchers related to data sharing, however, for every concern there is a feasible solution (Figure 1):

*Data sharing requires considerable work and there is currently little credit for data sharing*—Data collection takes considerable time and effort to assure that the quality of the data is high and the data is properly cleaned and used appropriately. Researchers are evaluated not on data sharing, but rather based on publications and grants, thus there is less incentive to engage in the considerable effort necessary to make a dataset available for sharing. *Solution*: Creating a mechanism that provides credit for researchers who make their data available to other researchers. This could be via a similar metric as a weighted “h‐index” for data sharing (share‐index), weighted by the amount or type of data that are made available. Researchers who use the data would then provide a data citation for the shared dataset and these could be used for promotion, tenure, and metrics used in decisions for grant funding.

Since researchers currently receive credit for the number of papers, one current approach to receive credit for data pooling initiatives is through authorship. This form of credit is highlighted by the increase in publications with over 200 authors. While the large number of authors may dilute the impact of those authors contributing the most work, without rewarding via authorship would likely impair initiatives such as the ENIGMA consortium. With the high impact of the ENIGMA papers, most journals provide a mechanism for allowing a large number of co‐authors. However, separating the names into “authors” and “contributors,” while providing equivalent credit for both, would provide a mechanism to credit those authors who did most of the work. Currently crediting those authors who did most of the work or who play a major role in the consortium is accomplished via the order of the authors at the beginning and end of the author list.

It is also possible to cite datasets as well as data papers, that provide a description of the data and metadata within a dataset. There are a growing number of journals that welcome data papers, such as *Nature ‐ Scientific Data* and *GigaScience*, and thus creating a mechanism in which credit is received for citations of data papers or datasets could provide a mechanism to help foster data sharing. An example of making data open access for researchers, coupled with the data and metadata involves a multimodal 7‐Tesla study that includes structural, diffusion‐weighted, susceptibility weighted, and functional MRI while watching the movie “Forrest Gump.”

*Other researchers will scoop us with data that we collected*—Assuring that the quality of the data is high and the data is properly cleaned and used appropriately is time consuming, but extremely important. Data sharing requires that those who use the data have a good understanding of the key variables, including the nuances of the data. If the data is released relatively quickly after collection, other groups may access the data and publish prior to those who actually have collected the data. Junior investigators are especially at risk, as they typically need more time to analyze and write the papers. They also may be busy curating other data or involved in course work, which would slow down the process of publishing. If a different group publishes first, it may make it difficult for the PhD student to publish, and thus could hamper their career. *Solution*: Providing a relatively short “grace period” that allows the researchers to publish initial studies with the data could resolve this issue.

*I don't have the financial resources needed for data sharing*—Preparing data for sharing is work above and beyond what is needed for a group to analyze data locally. From the perspective of a research group, this time could be spent doing other tasks important to completing the research project or for obtaining additional funding. Thus, there is little motivation for many researchers to take the time and effort to engage in data sharing. *Solution*: Funding agencies should both reward those who make the effort to share data and provide extra support that covers the costs of the work, storage, and support involved in data sharing.

*The data can be easily used to re‐identify the individual*—In some cases, for example, rare disease, or an extremely high‐profile scientific focus, the risk level may be too high for sharing. *Solution*: In this case one can still share coordinates or group level maps, or use a decentralized approach such as COINSTAC.

*I'm afraid my data can be used for unintended purposes*—Data misuse can occur at different levels. An extreme example would be that data is leaked to health insurance companies. *Solution*: A valid DUA and Data Transfer Agreement (DTA), based on the laws of the country of the researchers, should help prevent the possibility of further distribution. However, there is always a risk and thus minimizing the risk, while promoting the advancement of scientific discovery is the goal. Attorneys whose job is to protect the university may side on the being overly risk aversive, limiting the risk to a university, while at the same time potentially limiting the advancement of scientific discovery. Thus, both teamwork and creating a risk/benefit balance is necessary and these may differ per institution.

While there are obstacles to data sharing, most of these can be overcome. Changing some of the obstacles would likely require changes in policies of funding agencies and journals to provide support and credit for those who make the time and effort to participate in data sharing. For additional references and resources related to data sharing, we point the reader to the following articles: Poline et al., 2012; Keator et al., 2013; Poldrack et al., 2014; Gibaud, 2011; Temal, Dojat, Kassel, and Gibaud, 2008; Jack et al., 2008; Zou et al., 2005; and Van Essen et al., 2012.

## 9 THE ETHICS OF DATA SHARING

There are many challenges relating to the sharing of neuroimaging data, of which each could be a paper in and of itself. One of the challenges for data sharing includes navigating data sharing initiatives within the ethical and the changing legal tides related to human subjects data. In light of recent legal changes, notably in the EU, ethical aspects related to data have actually become more straightforward. These laws, in essence, give much of the control back to the participants. Within this context, the most important aspect of data sharing is obtaining thorough and transparent informed consent and when appropriate, informed assent. Research studies involving neuroimaging require approval from the local medical ethics committee or institutional review board in accordance with the Declaration of Helsinki (WMA, 2000). The consent form should provide an overview of the goals of the study, how the data will be used, a general description of who will have access to the data (academic institutions, industry, etc.), how long the data will be stored, and safeguards for data security. Further, data use or DTAs should be created to adhere to the laws of the country where the data has been collected. Those who sign the DTA or the DUA must agree to abide by the laws regarding the use of the data from the country which the data has been collected.

All human subjects data that is shared should have all personal identifiers removed and data that is not already open to the public should be stored both locally and at the site where the data is shared behind protected firewalls. If the data is analyzed external to these settings, it should be on a securely encrypted drive.

### 9.1 Data sharing and the GDPR

Those who are living in Europe are well acquainted with changes in data as a result of the GDPR. The GDPR was implemented on the 25th of May 2018 to provide data protection regulations for the inhabitants living or traveling within the EU. For data sharing of human subjects data both within and outside of the EU, the GDPR requires that specific information be provided on both participant information forms and consent forms. Explicit information regarding how the personal data will be used, for how long it will be used, who will have access to the data (i.e., researchers, industry), whether the data will be shared in a de‐identified manner should be provided in plain language to the participants. Moreover, the consent form should specifically ask for consent to share data with countries that have both similar and less strict privacy protection policies than the EU. Specific rules apply for data sharing with countries with similar privacy protection, (i.e., countries that fall under the GDPR adequacy decision [Council of the European Union and European Parliament, 2016]), and thus to share data with other countries, additional safeguards are often necessary.

### 9.2 Data sharing and the HIPAA

For those living in the United States, most human subjects research falls under the “Common Rule” (45 C.F.R. § 46 Subpart A), which is based on the 1975 revision of the Declaration of Helsinki. However, research taking place with personal health information from covered institutions (i.e., hospitals, clinics, etc.) falls under the HIPAA. HIPAA was implemented in 1996 and the “Privacy Rule” was incorporated April 14th, 2003. The most notable difference between the GDPR and HIPAA is to whom the regulations apply. The GDPR applies to anyone who is processing personal data within the EU and anyone outside the EU processing personal data from individuals within the EU. HIPAA applies to covered entities only, covered entities are health plans, health care clearinghouses, and health care providers electronically transmitting health information in connection with transactions for which Health and Human Services (HHS) has adopted standards (45 C.F.R. § 160.103). Research involving PHI from non‐covered institutions does not fall under HIPAA, but rather under the Common Rule. As certain institutions have both covered and non‐covered functions, there is a possibility to elect for being a hybrid entity, where only the covered functions must comply with the HIPAA requirements under the Privacy Rule. PHI not held by a covered entity can be used and disclosed without regard to the Privacy Rule. However, specific state regulations such as the “Federal Policy for the Protection of Human Subjects” or the Common Rule still apply.

For data sharing within and outside the United States, HIPAA does offer opportunities for sharing with researchers. For example, clinical neuroimaging data is held by covered entities and HIPAA applies to this data. Covered entities are permitted to share PHI without individual consent if (a), a waiver of authorization for the disclosure of PHI is approved by the IRB; (b), with confirmation by researchers that they will use the data only to prepare a research protocol or for similar purpose preparatory to research and the researcher will not remove PHI from the covered entity and that the data is necessary for the study; or (c), with representations of the researcher that data will be used only for research on the PHI information of decedents and the data is necessary for the study and documentation of the death of the individual (45 C.F.R. § 164.512(i)). In addition, similar to the GDPR, the Privacy Rule also allows for research use, disclosure, and data sharing when consent is obtained from the participant (45 C.F.R. § 164.508). With the protection of a DUA limited datasets can also be shared to address specific research questions.

## 10 DISCUSSION

The last decade has seen a dramatic increase in data sharing, data pooling, and the formation of collaborative data harmonization and analysis networks, such as ENIGMA. The reason why these initiatives are gaining momentum is because they foster collaboration and can advance the pace of scientific discovery. Data sharing allows for greater transparency in science with the ability to promote reproducibility and replication of study findings (Button et al., 2013; Ioannidis, 2005; Open Science Collaboration, 2015). Data sharing is cost effective for funding agencies (Milham et al., 2018), as they are not funding redundant studies and thus they see “more bang for their buck.” In addition, data can be shared with investigators from low‐ and middle‐income countries who may not have the resources to conduct expensive neuroimaging studies, but do have the ability to ask interesting and creative questions of the data. Finally, if participants provide consent for their data to be shared, which is the most important element, then they can enjoy knowing that researchers across the globe are potentially working with their data to better understand the complexities of brain structure and function and to bring about novel discoveries.

### 10.1 Scientists without borders

The greatest obstacle to sharing medical research data is not because of the laws, but rather the researchers and the institutions. Scientists are sometimes not overly keen about sharing their data with others. There are very real issues related to data sharing that make researchers less willing to share. The most common is that it takes considerable effort to collect and collate the data and others could then publish results sooner than those who actually collected the data. Providing some time for those who collected the data to write up the results, however, can typically circumvent this issue. Further, some studies may be quite complex and those using the data may not fully understand the sometimes‐subtle complexities of the data. This can result in either misuse or the investigators who collected the data serving as a “help desk” for those using the data. Further, some studies have a different business plan in which data sharing is tied to monetary reimbursement to help support further work or data collection by the researchers.

However, we believe that scientists should be at least as altruistic as the participants who are participating in their research studies. While it may appear that laws, such as the GDPR are in place to limit data sharing initiatives, this is far from the truth. The goal of the GDPR is to provide greater control and protection to the individual over whose data has been collected. Thus, the key issue is to offer the opportunity for the participants to share their data, if they would like. This can be done through obtaining consent for data sharing (and assent when appropriate) that adhere to the regulatory laws of the country of the study. Researchers should strongly consider that the participants are provided the option whether they want their data shared with other researchers. Further, it is often the case that researchers who utilize shared de‐identified human research data will need to sign DUAs that adhere to the laws specific to the country of the participants (i.e., GDPR for data shared from the EU, or the DUA to use ABCD Study data from NIDA). Within the EU and likely other countries, the complexities of these laws and the fears of retribution may serve as a rationale for some researchers for not sharing. However, with proper consent and, when necessary, completing data use or DTAs, sharing data in most countries, including the EU, is possible.

### 10.2 Scientists within borders

There is no question that data sharing will entail some level of risk. A data leak of sensitive information, for example, could result in individual's data being used for unintended and potentially harmful purposes. However, if all researchers kept their data under tight control with no data sharing outside their research group, this will hamper the progress of scientific discovery. Thus, there is a balance. Not only should strict precautions be set to assure to the best means possible the protection of individual data, but there needs to be some level of risk/benefit ratio for data sharing. Different countries may differ slightly in the level of restrictions towards data, however, these differences become equalized to some extent if the participants are allowed to decide how they want their data to be used.

Attorneys who work for specific universities have the goal to assure that the university is protected from potential legal actions, such as the potential 20 million euro fine imposed by non‐compliance with the GDPR. This may result in the setting of a very restrictive bar for individual researchers within certain institutions. Thus, within this framework, it is very important that the research community work together with attorneys and ethicists to determine what is necessary with respect to making important advances in medical research while offering adequate protection for human subjects data. Within the field of bioinformatics, mechanisms are emerging that allow for data sharing without the data ever leaving the institution where the data collected (Landis et al., 2016), which can offer the opportunity for institutions with more restrictive policies to be able to engage in data sharing initiatives.

## 11 CONCLUSIONS

Collaborative networks and data sharing initiatives are broadening the opportunities for the advancement of science and the ability to ask important research questions that could benefit others. These initiatives offer greater transparency, with the opportunity for external research groups to reproduce and/or replicate findings (Nichols et al., 2017). There are both real and imagined obstacles for data sharing which equate with not all researchers being supportive of data sharing initiatives. For researchers who are not keen on data sharing, recent and emerging regulations regarding human subjects data can be used as a barrier, or excuse, for not taking part in data sharing initiatives. However, there should be a balance, as keeping data under lock and key for use by only a handful of researchers may protect privacy, but will limit scientific discovery. Alternatively, sharing everything with everyone does not safeguard individual privacy. The safeguarding and ethical use of that which has been entrusted to us (data) is the responsibility of all researchers, irrespective of the GDPR, HIPAA and other regulations that exist. While we do not intend to minimize the importance of data security, there is a certain fear that has emerged regarding data sharing where it has become greater than life, monsters under the bed. We have provided approaches to neuroimaging and metadata that can help protect the privacy of the research participants involving data sharing initiatives. However, one key element that is often not discussed in regards to data sharing is the wishes of the participant in allowing their data to be shared.

Researchers can provide the opportunity for the participants to decide whether they are willing, or would like that their data be shared. This can take place via discussions with the participants and providing information and the choice on the consent form. The researchers should then set up the proper safeguards under the law to both protect the data to the greatest extent possible, while also sharing the data if that is the wish of the participant. Then, within the context of contentiously obtaining consent and the use of proper data use and DTAs, if legal cases are brought against a researcher or an institution, then it is open science that will be brought to trial, which is a battle worth fighting for.

## Data Availability

Data sharing is not applicable to this article as no new data were created or analyzed in this study.
