# Correction to “Data Sharing and Privacy Issues in Neuroimaging Research: Opportunities, Obstacles, Challenges, and Monsters Under the Bed”

**DOI:** 10.1002/hbm.70335

**Published:** 2025-08-28

White T. , E. Blok , and V. D. Calhoun . 2022. “Data Sharing and Privacy Issues in Neuroimaging Research: Opportunities, Obstacles, Challenges, and Monsters Under the Bed.” Human Brain Mapping 43, no. 1: 278–291. 10.1002/hbm.25120.32621651 PMC8675413

The word ‘contentiously’ in the last sentence of the article should have been ‘conscientiously’' The last sentence of the article should thus read:

“Then, within the context of conscientiously obtaining consent and the use of proper data use and DTAs, if legal cases are brought against a researcher or an institution, then it is open science that will be brought to trial, which is a battle worth fighting for.”

We apologize for this error.

